# Bioremediation of Lubricant Oil by Environmentally Adapted *Pseudomonas aeruginosa*, *Pseudomonas putida*, and *Proteus vulgaris* in Houston, Texas

**DOI:** 10.3390/biotech15020027

**Published:** 2026-03-26

**Authors:** Sadith Mosquera, Jason A. Rosenzweig

**Affiliations:** 1 Department of Environmental and Interdisciplinary Sciences, College of Science, Engineering and Technology, Texas Southern University, Houston, TX 77004, USA; s.mosquera3973@student.tsu.edu; 2 Department of Biology, College of Science, Engineering and Technology, Texas Southern University, 3100 Cleburne Street, Houston, TX 77004, USA

**Keywords:** toxicants, watersheds, pollutants, opportunistic pathogens

## Abstract

Lubricating oil (LO) is manufactured in various formulations for different applications. The inappropriate disposal of petroleum hydrocarbons can increase soil contamination, promoting deleterious environmental and human health impacts. More specifically, following prolonged exposure, LO contaminants are known to have carcinogenic and neurotoxic effects in humans. Bioremediation provides an effective and attractive strategy to expedite the clean-up processes of LO contaminants. We isolated and identified environmentally adapted strains of *Pseudomonas aeruginosa*, *Pseudomonas putida*, and *Proteus vulgaris* from Houston watershed bayou soils. Interestingly, all three exhibited increased resistance, vis-a-vis surrogate strains, to various antibiotic challenges (of chloramphenicol, tetracycline, kanamycin, penicillin, streptomycin, etc.) and increased biofilm formation ranging from 1.6 to 6.7-fold. In fact, all three environmental strains were significantly better at producing enhanced biofilm formation in the presence of spent LO rather than clean LO as well as outproducing biofilm made by the surrogate strains. Finally, the environmental isolates *P. aeruginosa*, *P. putida*, and *P. vulgaris* demonstrated an enhanced ability to sequester clean (2-, 2.5- and 1.14-fold) and spent (1.4-, 1.5, and 1.2-fold) LO when compared to their commercially acquired surrogate reference strains. Our three environmentally isolated organisms from Houston watershed soils appeared to be environmentally adapted to tolerate LO exposures. In the presence of LOs, all three environmentally isolated strains exhibited enhanced growth, enhanced biofilm production, and improved bioaccumulation of LOs relative to commercial reference strains. Taken together, environmentally adapted organisms can promote the bioremediation of contaminants threatening our environment and, potentially, human health.

## 1. Introduction

Lubricating oil (LO) is widely used for multiple applications, including the lubrication of internal combustible engines [1]. In that vein, a significant portion of LO is utilized in automobile engines, where it reduces wear and tear by minimizing friction and protects against rust [2,3]. Locally, in 2022, the U.S. Department of Transportation reported 3,493,246 registered cars in Harris County, Texas (which includes Houston). As a result, extensive use of LO will inevitably lead to increased environmental pollution via spills, leaks, and inappropriate/illegal disposal. More specifically, it is estimated that approximately 50% of LOs are disposed of into the environment [4] causing extensive harm to various ecosystems. Moreover, the entire biosphere is polluted by between 10 and 15 million tons of LO and their derived petrochemicals each year, mainly sourced from industrial processes [5].

As one of the leading oil-producing states in the United States, Texas has unfortunately experienced significant numbers of oil spills from pipelines, production facilities, and transportation systems. More specifically, the Mega Borg spill in 1989 released 5.1 million gallons of oil into the Gulf of Mexico and was partially remediated using oil-degrading bacteria [6]. More recently in 2010, the Deepwater Horizon oil well explosion catastrophically released approximately 210 million gallons of oil into the Gulf of Mexico, making it the largest marine spill in U.S. history [6]. Additional accidents include: the Texas City Refinery spill in 2014, which discharged thousands of barrels of oil into Galveston Bay [7], the 2015 Houston Ship Channel collision [8], and the Magellan Pipeline rupture in 2017 [9]. Most recently, a Houston-based company leaked 3500 gallons of LO into an industrial canal in Lake Charles, Louisiana [10]. Taken together, the frequency and scale of oil-related accidents in Texas, the Houston region, and its surrounding area highlight the need for effective management and mitigation strategies.

As mentioned earlier, environmental persistence of LO poses significant ecological and human health risks. Beyond major industrial spills, improper disposal of lubricating oil and unsafe dumping of used filters and containers by unscrupulous individuals can also contribute to LO toxin accumulations and exacerbate soil contamination [11]. Spent LO from various combustible motors tends to be more readily bioremediated than clean LO on account of having a mixture of minerals including calcium, and metallic contaminants [12]. This presents a challenge in environments contaminated with both clean and spent LOs. Compared to clean LO, spent LO typically contains higher levels of polycyclic aromatic hydrocarbons (PAHs), making them more toxic than clean LOs [13]. PAHs have been strongly linked to carcinogenic, mutagenic, and neurotoxic effects in humans and animals [11,14]. In addition to soils, aquatic ecosystems are particularly vulnerable to LO contamination. Ultimately, both aquatic and soil LO contaminations damage food chains, threaten human food security, and can devastate surface and groundwater quality [15]. To address this directly, bioremediation technologies can work to ameliorate contaminated soils and waters as an adjunctive application when coupled with other remediation approaches.

Bioremediation has emerged as one of the most attractive, cost-effective, and eco-friendly strategies to remediate soil and water contaminated with LOs. As compared to less economical and inefficient physical or chemical remediation methods, microbial bioremediation exploits natural microbial metabolic pathways that degrade the hydrocarbons in LOs into less harmful byproducts [16,17]. One approach is bioaugmentation, the introduction of specialized microbial strains into contaminated environments for the purpose of bioremediation [18]. Another approach is bio-stimulation which enhances the activity of indigenous microbial communities, native to the environment, through prompted nutrient or environmental management [18,19]. *Pseudomonas* spp., *Proteus* spp., and *Bacillus* spp., have all shown remarkable hydrocarbon-degrading capabilities [20,21]. Furthermore, these properties can be enhanced through biosurfactant production, which increases the bioavailability of LOs [22,23] Evaluating the bioremediation potentials of indigenous/local isolates that often grow and even thrive in LO-polluted soils is critical for optimizing the bioremediation of Houston area contaminated soils. In this study, we aimed to isolate and identify Houston soil bacteria capable of metabolizing clean and spent LOs, and we hypothesized that our environmentally isolated organisms would be better adapted than commercially acquired reference strains at sequestering LOs and producing biofilm (which has been shown to help concentrate bacterial biomasses). Our goal is to help advance bioremediation strategies for oil-contaminated soils. Notably, we observed that environmentally isolated *P. aeruginosa*, *P. putida*, and *P. vulgaris* demonstrated an enhanced ability to sequester clean and spent LO, were better adapted to withstand antibiotic challenge, and exhibited increased biofilm production.

## 2. Materials and Methods

### 2.1. Bacteriological Medium, Surrogate Strains, and LOs

M9 minimal broth and agar media (AMRESCO LLC Solon, OH, USA cat. number J863-500G) was used for the cultivation of both environmentally isolated and commercially acquired surrogate strains. M9 minimal medium is a highly referenced microbial growth medium [24]. that contains only salts and nitrogen, so it is traditionally supplemented with glucose, amino acids and vitamins as needed. For this study, glucose was eliminated, and LO was added as the sole source of carbon.

Surrogate strains of *Pseudomonas aeruginosa* (Carolina cat # 155250A), *Pseudomonas putida* (ATCC cat # 12633), and *Proteus vulgaris* (Carolina cat # 155240A) were purchased for comparative studies. Around 500 mL of spent LO was obtained from a 2000 Ford-150 pickup truck at the Walmart located 2391 S Wayside Dr., Houston, TX 77023, USA. Castrol GTX 10W-40 was used as the clean LO in these studies. The compositions of the spent and clean LOs were not determined.

### 2.2. Soil Sample Collection and Bacterial Isolation

Soil sampling was carried out using our previously published protocol [25]. with some modifications. In short, soil samples collected in triplicate at a distance between 30 and 50 cm from two bayous within the Houston area, the Buffalo Bayou (29.77, −95.44—a heavy populated watershed) and Carpenters Bayou (29.84, −95.16—a rural, low population watershed), following EPA standards. In short, samples were collected from a topsoil depth between 0 and 15 cm using a stainless-steel soil auger and trowel. The three samples were mixed thoroughly within polyethylene bags (zip lock bags) to constitute a composite sample from the site, transferred into a cooler maintained at 4 °C, and transported (1 h) to Texas Southern University, where upon arrival pH and total solids in each sample were measured (Table 1). Finally, samples were stored at 4 °C for further analysis. For bacterial isolation, a modified protocol of Refs. [26,27] was employed. In short, 2.5 g of soil was added to 50 mL of M9 medium containing either 0%, 5%, 10%, or 20% (*v*/*v*) clean or spent LO. The concentrations were selected based upon the fact that soil hydrocarbon contaminations depend upon several factors, including (but not limited to) proximity to source, etc. As a result, contamination concentrations can range anywhere from 0.1 to 6%, even in some cases going as high as 10% [28]. Therefore, we chose 5, 10, and 20% to cover the environmentally relevant range observed in some affected soils following a spill. Samples were thoroughly mixed and incubated at 30 °C with orbital shaking (150 rpm) for 7 days.

### 2.3. Enumeration of Soil Bacteria Isolates

Following one week of incubation, 1 mL of the culture broth was serially diluted 10-fold, in triplicate, plated on 1.5% agar (Thermo Fisher Scientific, Waltham, MA, USA, A360-500) M9 plates pre-treated with 400 μL of either clean or spent LO, and incubated at 30 °C for 5 days. Colonies were enumerated by direct plate counting, differences in phenotype/colony morphology were noted, and down-selected colonies were sub-cultured on either Luria–Bertani agar (Thermo Fisher Scientific BP1426) or MacConkey agar (Thermo Fisher Scientific R453802). This step was repeated twice to obtain purified strains for downstream Gram-staining and ribotyping.

### 2.4. Ribotyping

Full-length 16S rDNA of the down-selected environmentally isolated colonies were PCR-amplified using universal primers, as done before [29,30]. In short, the primer set of 27F (AGAGTTTGATCCTGGCTCAG) and 1387R (GGGCGGGTGTACAAGGC) was used together with bacterial colony isolate lysates, and a Taq quick-load Master Mix Polymerase (New England Biolabs cat # M0486S, Ipswich, MA, USA) was used to generate the ~1.3 kb amplicons using a Bio-Rad T100^TM^ thermocycler (Bio-Rad Laboratories, Hercules, CA, USA). Cycling steps and temperatures were as follows: an initial denaturation at 95 °C for 5 min, a subsequent denaturation at 95 °C for 1 min, a primer annealing temperature of 55 °C for 1 min, an extension at 72 °C for 1 min, and a final extension at 72 °C for 5 min. After preparing a 0.7 agarose gel, 1/5 of PCR amplicons were resolved and visualized with Syber Safe (Invitrogen cat # S33102, Waltham, MA, USA) staining and submitted for sequencing. Sequencing of the 16S amplicons using the 27F primer was performed by Lone Star Lab (Houston, TX, USA) using Sanger sequencing. In short, samples were cleaned with a G50 sephadex column (Cytiva, Marlborough, MA, USA) and 10 µL of PCR amplicon reaction templates and 5 µM of the 27F primer were used for sequencing reactions. Further, within each reaction 4 µL dGTP BigDye (Thermo Fisher Scientific, Waltham, MA, USA) and 4 µL of 5× buffer per reaction was used. The PCR cycling conditions included a preheat of 95 °C for 60 sec followed by 48 cycles of: (1.) 95 °C for 30 s, followed by (2.) 50 °C for 15 s, and finally (3.) 60 °C for 120 s. Once complete, samples were cleaned once more using a G50 96 well-sephadex plate, were run through an ABI 3730XL sequencer (Thermo Fisher Scientific, Waltham, MA, USA), and data were analyzed. Then, 16S rDNA sequences were subjected to an NCBI BLAST + 2.17.0 for comparison to all sequences that have been deposited in the GenBank database.

### 2.5. Growth Kinetics

For growth kinetic assays, we adopted our previous protocol [31,32,33]. In short, bacterial isolates and their respective surrogate strains were grown to saturation and then diluted to a starting 0.2 OD_600nm_ in a 96-well plate (200 μL/well) supplemented with clean or spent LO at 0%, 5%, 10%, and 20%. Plates were shaken 2 min prior to OD read to allow for aeration and mixing of samples. Growth was monitored every 60 min for 19 h at 600 nm. All growth assays were conducted in triplicate.

### 2.6. Antibiotic Susceptibility Testing and Cold Growth

To evaluate whether our environmental isolates exhibited increased antibiotic resistance in the presence and absence of LO, we employed the Kirby–Bauer disk diffusion assay [34] with some modifications. Bacterial cultures of approximately 0.5 (OD_600nm_) were plated on 5 mm thick LB agar plates, normalizing for biomass of each strain. Chloramphenicol (30 µg), tetracycline (30 µg), kanamycin (30 µg), penicillin (10 IU), streptomycin (10 µg), novobiocin (30 µg), erythromycin (15 µg), and neomycin (30 µg) impregnated disks were placed equidistantly on the inoculated plates using ethanol sterilized forceps to determine zones of inhibition (diameters were measured in mm). Reported zones of inhibition were compared to Clinical and Laboratory Standards Institute (CLSI) thresholds for *Enterobacteriaceae* resistance determinations (for *P. vulgaris*) following 18 h of growth on LB agar plates. To determine if cold temperatures influenced bacterial growth in the presence of ~2% LO, 400 µL of either clean or spent LO were spread on the surfaces of 20 mL LB agar plates, and plates were incubated at either 25 or 32 °C for 18–24 h. The temperatures were chosen to reflect water temperatures typically recorded in Houston watershed soils throughout the year.

### 2.7. Biofilm Formation Assay

For biofilm assays, our previous published protocol was followed [31,32,33] with some modifications. In short, reference and environmental isolates grown in M9 medium supplemented with clean or spent LO at 0%, 5%, 10%, and 20%, were diluted to 0.2 OD_600nm_ in a 96-well plate (200 μL/well), and microtiter plates were incubated for 24 h with agitation (∼100 rpm) at 37 °C. Next, biofilm biomass was quantified after wells were washed with water and incubated with 0.1% (*v*/*v*) crystal violet (125 μL/well) for 1 h at room temperature. Unbound crystal violet was removed by a water wash, and wells were allowed to air dry overnight. Biofilm-bound crystal violet was dissolved in 250 μL of 30% acetic acid, and solubilized crystal violet was measured at OD_570nm_. Biofilm production was normalized based on relative biomass (optical densities of biofilm formed/optical density of terminal bacterial growth), and all experiments were carried out in triplicate. Non-linear relationships between biofilm production and growth rates were not characterized in this study. Additionally, OD_600nm_ reads could be influenced by both LO emulsification and demulsification, particularly by *P. aeruginosa* which produces biosurfactants [35].

### 2.8. Extraction and Quantification of Residual Oil

Following bacterial growth in either 1% clean or spent LO for 7 days, the aqueous layer of the spent M9 medium was carefully removed and filtered through Whatman No. 1 filter paper (Thermo Fisher Scientific 09-805-1B). The filtration of the aqueous layer removed biomass and cell debris. The residual oil retained on the filter paper was extracted with 5 mL volume of dichloromethane (CH_2_Cl_2_) (Lab Alley Essential chemicals lot # 5000621/1.1, Austin, TX, USA). Like chloroform, LOs are highly soluble in CH_2_Cl_2,_ and 80% recovery rates are possible. The remaining contents in the flask were further extracted in 5 mL of CH_2_Cl_2_ and then transferred onto the filter paper. Combined filtrates of both extractions were collected in a clean, dry, and pre-weighed grease-free glass vial; the oil–solvent mixture was evaporated to a constant mass on a hot plate at 80 °C. The quantity of the undegraded residue was calculated as a percentage (in grams) of the amount of oil recovered from the sterile uninoculated control normalized to 100% [20,36]. LO sorption on the biomass was not evaluated.

### 2.9. Statistical Analysis

All experiments were carried out in triplicate and averaged. Statistical analysis of the data was obtained using a two-tailed Student’s *t*-test (unequal variance). Significant differences were considered with *p*-values ≤ to 0.01 (**). Bonferroni corrections were made for pairwise *t*-tests for multiple comparisons.

## 3. Results

### 3.1. Environmental Bacterial Loads and LO Isolates

**Figure 1:**
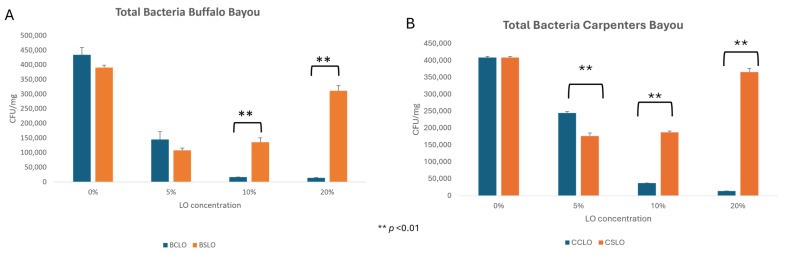
Houston watershed soil bacterial counts in the presence of clean and spent LO. Soil bacterial loads from Buffalo (**A**) and Carpenters Bayous (**B**) were exposed to either clean or spent LO at concentrations of 0, 5, 10, and 20%, and comparisons between the two were made. The experiment was performed in triplicate, and both standard error and statistical significance are shown (based on Student’s *t*-test with Bonferroni correction; α < 0.0125). BCLO = Buffalo Bayou clean lubricating oil, BSLO = Buffalo Bayou spent lubricating oil, CCLO—Carpenters Bayou clean lubricating oil, and CSLO—Carpenters Bayou spent lubricating oil.

It is approximated that between 90 and 95% of environmental microbes are unculturable, and it is possible that some of those unculturable microorganisms could be contributing to LO bioremediation. However, for this study we sought to focus on representative culturable microorganism contributions to bioremediation. More specifically, to determine whether environmentally adapted culturable bacteria from the urban Buffalo Bayou were better suited to grow in the presence of LO exposures than culturable bacteria from the more rural Carpenters Bayou, soil bordering both bayous was collected. In the Buffalo Bayou, significant reductions in bacterial loads were observed when soil bacteria were challenged with both clean and spent LOs compared to untreated soil samples (Figure 1, Panel A). Following the 10% and 20% spent LO challenge, there were significantly higher bacterial loads measured than in soil loads challenged with the same percentages of clean LO (Panel A). In contrast, and for reasons unclear, the 5% clean LO challenge yielded higher bacterial loads than the spent LO challenge, although not significantly (Panel A).

Surprisingly, in the more rural Carpernters Bayou, the same trends were observed. More specifically, the 5% clean LO challenge yielded significantly higher bacterial loads than the 5% spent LO challenge (Panel B). However, as was observed for the urban Buffalo Bayou soil communities (Panel A), following a spent 10% and 20% LO challenge, there were significantly higher bacterial loads measured than in those soil loads challenged with the same percentages of clean LO (Panel B). These data show that Buffalo Bayou soil bacterial communities from the urban core were no better adapted to tolerate spent LO contaminants than those bacterial communities in the more rural Carpenters Bayou setting.

In total, 52 isolates were identified in the following distribution: 19% *Pseudomonas* spp., 15.4% *Acinetobacter* spp., and 6% *Brucella* spp., *Stenotrophomonas* spp., and *Proteus* spp. (Table 2). Down-selected colonies were identified via ribotyping, and *Pseudomonas aeruginosa* (Carpenters Bayou), *Pseudomonas putida* (Buffalo Bayou), and *Proteus vulgaris* (Buffalo Bayou), were chosen for down-stream assays based on their being either opportunistic human pathogens (in the case of *P. aeruginosa* and *P. vulgaris*) or well-documented bioremediators of hydrocarbons (in the case of *P. putida*). We then sought to determine whether any of the three aforementioned environmentally isolated organisms were better adapted to tolerate LO contaminants than their commercially acquired surrogate strains.

### 3.2. LO-Influenced Growth Kinetics

**Figure 2:**
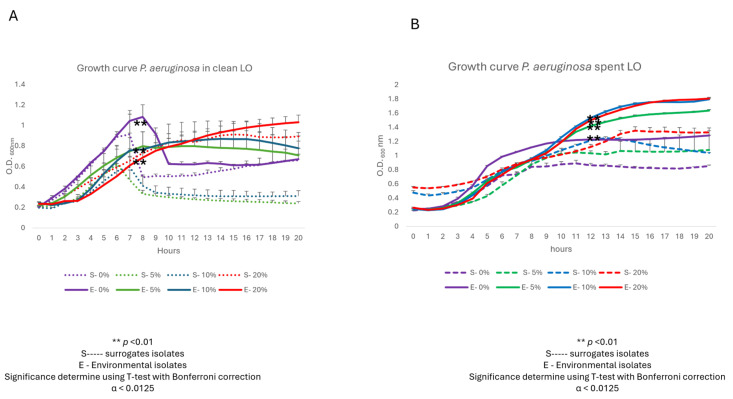
Growth response of environmentally isolated and surrogate *P. aeruginosa* strains in the presence of clean (**A**) and spent (**B**) LO. This experiment was performed in triplicate, and Student’s *t*-test (with Bonferroni correction; α < 0.0125) was used to determine statistical significance.

With clean LO as the sole carbon source, the environmentally isolated *P. aeruginosa* grew significantly better than its surrogate strain at 5 and 10% challenges; however, for reasons unclear, at the 20% clean LO challenge the surrogate strain improved its growth (Figure 2A). Similarly, when spent LO served as the sole carbon source, the environmentally isolated *P. aeruginosa* grew significantly better than its surrogate strain at 5, 10, and 20% challenges (Panel B). As was seen with clean LO (Panel A), the surrogate strain grew best at 20% challenge, albeit significantly lower than the environmental isolate (Panel B). Additionally, the highest biomass observed was by the environmental isolate grown in spent LO at 20 and 10% concentrations followed by 5%, all significantly higher than the surrogate strain grown at the same spent LO concentrations (Panel B); notably, the largest biomass was observed at 20% spent oil, with OD_600nm_ values approaching 1.8 by 18 h, nearly double the peak seen in clean LO (Panel B vs. Panel A). This indicates a stronger metabolic response, possibly due to the presence of partially degraded hydrocarbons or enriched nutrient profiles in spent LO. While the reference strain exhibited improved growth in spent LO compared to clean LO, particularly at 10% and 20% concentrations, its growth remained significantly lower than that of the environmental strain across all conditions (*p* < 0.01; Panel B). Taken together, these data suggest that the environmentally isolated *P. aeruginosa*, an opportunistic human pathogen, from the Houston watershed was adapted to grow in the presence of spent LO toxicants and even potentially utilizes components as nutritional substrates.

**Figure 3:**
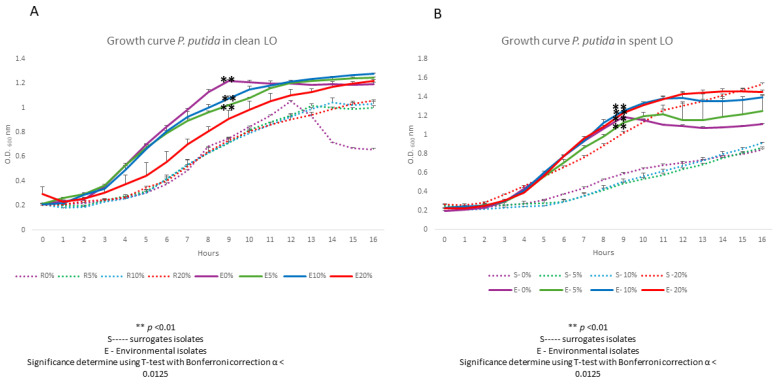
Growth response of environmentally isolated and surrogate *P. putida* strains in the presence of clean (**A**) and spent (**B**) LO. This experiment was performed in triplicate and Student’s *t*-test (with Bonferroni correction; α < 0.0125) was used to determine statistical significance.

As was expected, our environmentally isolated *P. putida* strain closely mirrored the growth of its surrogate strain initially at all clean LO concentrations tested, i.e., 5, 10 and 20% (Figure 3A). Growth began to diverge significantly between 4 and 12 h, with the environmental isolate entering the exponential phase earlier and reaching a higher stationary phase OD (Panel A). Also, both strains’ final biomasses were much higher than those of *P. aeruginosa* in clean LO (Figure 3A vs. Figure 2A). Further, in spent LO, biomass was significantly higher for the environmental strain vis-a-vis its surrogate strain at the at all time points, including the 16 h end point (Panel B). As a bona fide bioremediator capable of metabolizing hydrocarbons, our environmentally isolated *P. putida* strain was better adapted to metabolize components of the spent LO than its surrogate strain. Interestingly, and for reasons unclear, the surrogate strain did not grow as robustly as the environmental isolate in the 0% LO challenges.

**Figure 4:**
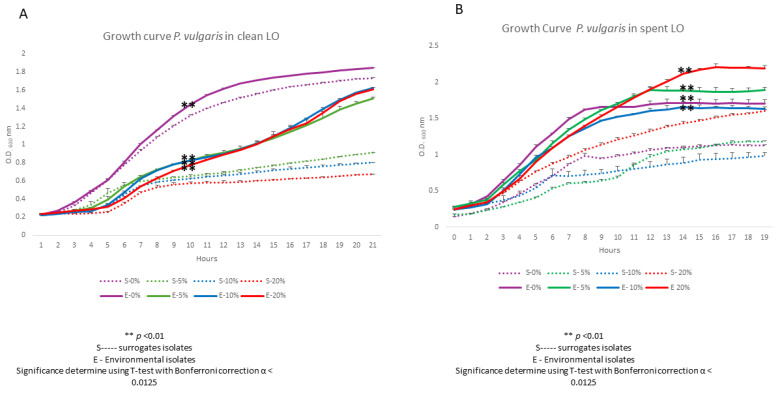
Growth response of environmentally isolated and surrogate *P. vulgaris* strains in the presence of clean (**A**) and spent (**B**) LO. This experiment was performed in triplicate and Student’s *t*-test (with Bonferroni correction; α < 0.0125) was used to determine statistical significance.

The *P. vulgaris* isolate significantly outperformed its reference strains across all clean LO concentrations tested from the 9 h midpoint until the 20 h endpoint (Figure 4A). More specifically, at 10 and 20% clean LO challenges, an OD_600nm_ reading near 1.4 at 19 h was recorded, suggesting no sensitivity or inhibitory effects of the clean LO (Panel A). When grown in spent LO at 5, 10 and 20% concentrations, growth was significantly higher than that of its surrogate form at the 8 h timepoint on (Panel B). Further, even higher biomass was achieved by the environmental isolate grown in spent LO than clean LO (Figure 3B vs. Figure 3A). For example, at 19 h an OD_600nm_ reading above 2.0 was recorded (Figure 3B). Taken together, the opportunistic pathogen isolated from the Houston watershed was adapted to grow in the presence of LO, with its most robust growth observed in the presence of 20% spent LO.

### 3.3. LO-Influenced Biofilm Production

**Figure 5:**
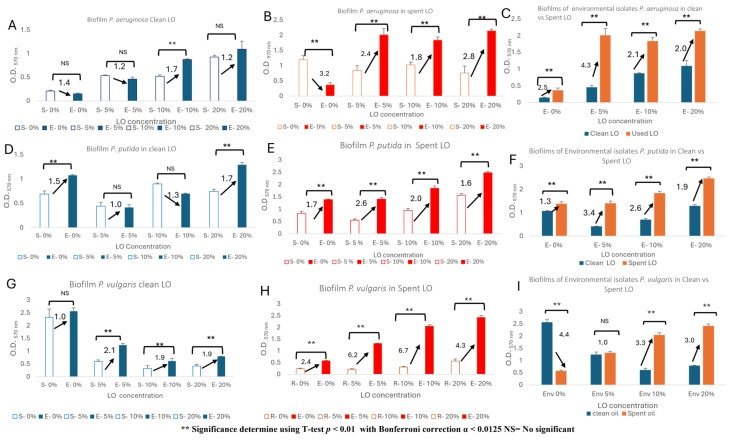
Biofilm production of *P. aeruginosa* (**A**–**C**), *P. putida* (**D**–**F**), and *P. vulgaris* (**G**–**I**) strains in response to clean (**A**,**D**,**G**) and spent (**B**,**E**,**H**) LO exposures. Both environmentally isolated and surrogate strains were exposed to 0, 5, 10, and 20% clean and spent LO. This experiment was performed in triplicate and Student’s *t*-test (with Bonferroni correction; α < 0.0125) was used to determine statistical significance.

Additionally, we sought to determine whether the environmentally adapted *P. aeruginosa*, *P. putida*, and *P. vulgaris* isolates exhibited enhanced biofilm production following LO exposures. Biofilm formation plays a critical role in environmental persistence and hydrocarbon degradation, as it enhances microbial survival under stress conditions and facilitates substrate degradation [37]. Our *P. aeruginosa* isolate produced significantly higher biofilm density than its surrogate strain when challenged with 10% clean LO and 5, 10, and 20% spent LO (Figure 5A,B). Furthermore, when comparing biofilm production of the environmental isolate in the presence of clean vs. spent LO, significantly higher biofilm was observed when grown in spent LO at 5, 10, and 20% concentrations (Figure 5C). With regards to the bioremediating *P. putida*, the environmental strain only produced significantly more biofilm during a 20% clean LO challenge (Figure 5D). As was expected, the environmental isolate produced significantly more biofilm than its surrogate when challenged with 5, 10, and 20% spent LO (Figure 5E) as well as exhibited significantly more biofilm when challenged with 5, 10, and 20% spent LO compared to biofilm production when challenged with the same concentrations of clean LO (Figure 5F). Unlike our other two environmental isolates, *P. vulgaris* produced significantly greater amounts of biofilm than its surrogate at 5, 10, and 20% clean and spent LO challenges (Figure 5G,H). However, the environmentally isolated *P. putida* only produced significantly more biofilm when grown in 10 and 20% spent LO compared to amounts produced when grown in clean LO (Figure 5I). Taken together, the *P. aeruginosa* isolates from Carpenters Bayou (a low population density) and the *P. putida* and *P. vulgaris* isolates from Buffalo Bayou (a densely populated area) demonstrated comparable LO bioremediation potentials and, possibly, similar environmental adaptations (relative to their surrogate strains).

### 3.4. LO-Influenced Alterations in Antimicrobial and Temperature Sensitivities

Beyond enhanced biofilm production and growth kinetics in the presence of LOs, we also sought to determine whether our environmental isolates were better adapted to resist antibiotics (another contaminant found in Houston watersheds; [38]) and temperature fluctuations, both important factors when considering their overall bioremediation potentials. With regards to antibiotic resistance, the environmental opportunistic pathogen *P. vulgaris* (isolated from the urban Buffalo Bayou) demonstrated resistance to all antimicrobials tested (when compared to its surrogate strain), with the exception to tetracycline (30 μg), to which it remined sensitive (Table 3). Unexpectedly, and for reasons unclear, the environmental *P. aeruginosa* displayed increased sensitivity to several antimicrobials tested (chloramphenicol, streptomycin, erythromycin, and neomycin) when compared to its reference strain (Table 3). The environmentally isolated *P. putida* exhibited both increased antimicrobial resistance (chloramphenicol, kanamycin, streptomycin, and neomycin) as well as no changes or even increased sensitivity (tetracycline, novobiocin and erythromycin) to other antimicrobials when compared to its surrogate strain (Table 3). Direct comparison of *Pseudomonas* spp. to CLSI standards are not applicable since the table presents only CLSI drug resistance standards for enteric bacteria, not pseudomonads. With regard to temperature, *P. aeruginosa* exhibited similar bacterial colony loads at either ~25 °C or 37 °C on nutrient rich medium (i.e., tryptic soy agar–TSA) supplemented with 2% ULO or SLO; however, when grown in SLO-supplemented, nutrient-poor M9 and minimal media, ~10X and ~2X increases in colony counts were observed following 37 °C vs. ~25 °C, respectively. For *P. putida*, greater colony numbers were observed in all growth conditions at 37 °C vs. ~25 °C, except M9 medium supplemented with SLO. *P. vulgaris* only exhibited increased colony numbers at 37 °C vs. ~25 °C when rich medium (TSA) was supplemented with either SLO or CLO. Taken together, it does appear that environmentally adapted bacteria do exhibit altered antimicrobial resistance and temperature-dependent growth profiles; however, not all profiles trend in the same direction within and among the various strains tested.

### 3.5. Bio-Sequestration

**Figure 6:**
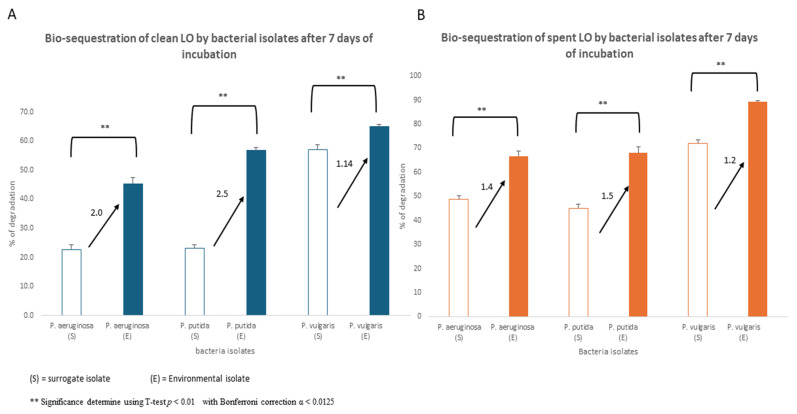
Bio-sequestration of clean and spent LO by environmental and surrogate *P. aeruginosa*, *P. putida*, and *P. vulgaris* strains. Both environmentally isolated and surrogate strains were exposed to 1% clean (**A**) and spent (**B**) LO. Following growth in LO for 7 days, residual oil within the medium was filtered out, extracted and quantified. Percentages of remining oil were calculated and reported. This experiment was performed in triplicate and Student’s *t*-test (with Bonferroni correction; α < 0.0125) was used to determine statistical significance.

Growth kinetics data suggested that our isolates were environmentally adapted and potentially metabolized components of LO (Figure 2, Figure 3 and Figure 4). To investigate this directly, we sought to evaluate *P. aeruginosa*, *P. putida*, and *P. vulgaris*’ bio-sequestration efficiencies of either 1% clean or spent LO following a 7-day incubation period. The environmental *P. aeruginosa* isolate sequestered ~50% of clean LO, compared to ~30% by the surrogate strain (Figure 6A). With regard to spent LO sequestration, the *P. aeruginosa* environmental strain demonstrated improved sequestration performance of 66% compared to the surrogate’s sequestration of ~45% spent oil (Panel B). Further, the environmental *P. putida* and *P. vulgaris* strains not only outperformed their isolates in the sequestration of both clean (~55% vs. ~22% and ~65% vs. ~55%) and spent LO (~65% vs. 45% and ~90% vs. ~72%), respectively, but also mirrored *P. aeruginosa* in that the greatest sequestration was observed in spent LO as compared to sequestration of clean LO for both organisms (Figure 6). As was expected for all three isolates, being environmentally adapted provided them with an apparent advantage in sequestering the hydrocarbons present in both clean and spent LO. Unexpectedly, however, and for reasons unclear, our environmentally isolated *P. vulgaris* was the best bioaccumulator as evidenced by the highest percentage bio-sequestration (Figure 6). This curious phenomenology warrants additional mechanistic studies to better explain *P. vulgaris*’ performance.

## 4. Discussion

According to the TCEQ, the Houston region recorded 460 used oil-related incidents and 923 violations from 2005 to 2025, reflecting persistent local challenges in preventing illegal disposal of LOs [39]. In this study, we sought to evaluate whether bacterial isolates, from Houston watershed soils, were adapted for enhanced growth, biofilm production, and antimicrobial resistance in the presence of LO toxicants as well as whether adapted strains exhibited increased biodegradation of LOs. In all experiments, we compared the effects of both clean and spent LO since clean LO primarily consists of hydrocarbons and chemical additives, whereas spent LO accumulates degradation by-products, heavy metals, and other toxic compounds during engine operation [13]. Both of the aforementioned LOs could find their way into the Houston watershed and associated soils via spills and unscrupulous, illegal dumping. Importantly, soil isolates of *P. aeruginosa*, *P. putida*, and *P. vulgaris* exhibited significantly higher growth potential in the presence of both used and clean LO than their respective surrogate reference strains. These findings suggest that adaptations to environmental toxicants could support bioremediation of environmental LO contaminants. Furthermore, environmentally adapted strains all exhibited increased antimicrobial resistance to a large number of antibacterial drugs, suggesting that environmental adaptation to watershed pollutants extended beyond LO. This raises unrelated concerns about the transmission of drug resistance among unrelated environmental bacteria within the Houston watershed [40]. It is possible that increased antimicrobial resistance and enhanced bioremediation of LO share genetic and/or metabolic pathways; however, such convergence, if any, would have to be worked out experimentally.

One mechanism that enables enhanced antimicrobial resistance and bioremediation of LO is increased biofilm production. More specifically, the biofilm structure prevents antimicrobial drugs from penetrating bacterial membranes. That, coupled with biofilm dispersion of antimicrobials, which lowers their effective concentrations, works to promote increased antimicrobial resistance [41]. In that same vein, bacterial biofilms promote enhanced bioremediation by immobilizing both the bacteria, which carry out the bioremediation, as well as the toxicants and pollutants. Doing so increases pollutant uptake and metabolization by the bacteria [37]. Viewed in this light, we sought to investigate whether exposure to LO promoted increased biofilm formation in our environmentally isolated bacteria. Interestingly, exposure to both clean and spent LO (at various concentrations) promoted significantly increased biofilm formation in all 3 of our isolates compared to surrogate reference strains. Therefore, our data shows that *P. aeruginosa*, *P. putida*, and *P. vulgaris* respond to increased LO exposure by robustly increasing biofilm formation, likely to prevent undesired toxicity as well as to concentrate the LO for enhanced biodegradation. Recognizing that *P. aeruginosa*, specifically, can produce a biosurfactant which can influence OD_600nm_ reads, the *P. aeruginosa* growth kinetic and biofilm formation data can be scrutinized a little more closely.

With regard to bio-sequestration, all three of our environmental isolates significantly outperformed their respective surrogate isolates. Unexpectedly, however, among the three tested isolates, the *P. vulgaris* environmental isolate surprisingly exhibited the highest sequestration rates, while *P. aeruginosa* and *P. putida* isolates carried out sequestration significantly better than their surrogate reference strain as well, albeit not as well as the *P. vulgaris* isolate. Although *P. putida* is more efficient than *P. aeruginosa* in sequestration of hydrocarbons and is a well-documented exemplary bioremediator of toxicants in general [42,43], in our study, the unsuspected *P. vulgaris* was the most efficient bioaccumulator of both clean and spent LO (~64% and ~90% biodegradation respectively). This phenomenon needs to be further investigated, and follow up studies will focus on sorption percentages, specifically in the biomass.

It is conceivable that the Houston watershed specifically selected for our *P. vulgaris* super-bioaccumulator of hydrocarbons based on the watershed’s toxicant composition. Further sequence analysis of the isolate’s genome may reveal clues about its unexpected and exceptional capacity for sequestration of hydrocarbons. Taken together, the complementary roles of different bacterial species in hydrocarbon breakdown and persistence in contaminated soil could be exploited as a bioremediation cocktail approach/strategy enabling complete, accelerated breakdown and neutralization of hydrocarbon toxicants/pollutants in soils. Further, the metabolic diversity and cooperative interactions of mixed cultures could extend degradation to a broader spectrum of oil components and toxicants (beyond just hydrocarbons), thereby ameliorating environmental conditions. Spent LOs typically contain poly aromatic hydrocarbons and metal contaminants that impede many biodegradation pathways; however, organisms typically recovered at such sites include: *Pseudomonas* spp., *Rhodococcus* spp., *Citrobacter* spp., *Serratia* spp., *Alcaligenes* spp., *Acinetobacter* spp., *Arthrobacter* spp., *Bacillus* spp., and *Micrococcus* spp. [44,45]. Following hydrocarbon degradation by the aforementioned organisms, sludges are produced by oxidation, and the entire process can take years [5]. Viewed in this light, employing native bacterial mixed cultures for local bioremediation provides a cost-effective and sustainable strategy as either a viable alternative to or an adjunctive treatment for chemical-based approaches.

In conclusion, our three environmentally isolated organisms from Houston watershed soils appeared to be environmentally adapted to tolerate LO exposures. In the presence of LOs, all three strains exhibited enhanced growth, enhanced biofilm production, and improved bioaccumulation of LOs relative to commercial reference strains. These findings suggest that when searching for solutions to bioremediation of polluted environments, one may not have to look farther than his/her own backyard, where local microorganism may already be environmentally adapted and ready for deployment.

## Figures and Tables

**Table 1:** Total dissolved solids (TDS) and pH values of soil samples.

Bayous	Coordinates	Total Dissolved Solids	pH
Buffalo Bayou	29.8–95.4	153 +/− 2.45	7.0
Carpenters Bayou	29.8–95.2	162 +/− 2.05	7.5

**Table 2:** *Bacterial library from Buffalo and Carpenters Bayous. Red highlighted strains were used in this study*.

Number	Location	Coordinates	Code	Bacteria Gram (-)PCR Identification
1	Buffalo Bayou	29.765180, −95.360479	BCLO 01	*Pseudarthrobacter oxydans 286/329 (87%)*
2	Buffalo Bayou	29.765180, −95.360479	BCLO 04	*Pseudomonas nitroreducens strain TSO25 145/162 (90%)*
3	Buffalo Bayou	29.765180, −95.360479	BCLO 5-1	*Ochrobactrum/Brucella cytisi strain HPG157 370/406 (91%)*
4	Buffalo Bayou	29.765180, −95.360479	BCLO 5-2	*Acinetobacter sp. strain 8637 255/301 (85%)*
5	Buffalo Bayou	29.765180, −95.360479	BCLO 5-3	*Acinetobacter baumannii strain FEA3 382/462 (84%)*
6	Buffalo Bayou	29.765180, −95.360479	BCLO 10-1	*Pseudomonas khazarica strain ODT-83 135/174 (78%)*
7	Buffalo Bayou	29.765180, −95.360479	BCLO 10-2	*Brucella thiophenivorans strain NK 382/447 (85%)*
8	Buffalo Bayou	29.765180, −95.360479	BCLO 20-2	*Proteus vulgaris strain BPGM7 112/128 (88%)*
9	Buffalo Bayou	29.765180, −95.360479	BSLO 5-1	*Acinetobacter sp. strain U17 247/282 (88%)*
10	Buffalo Bayou	29.765180, −95.360479	BSLO 10-3	*Acinetobacter pittii strain 2014S06-099 194/239 (81%)*
11	Buffalo Bayou	29.765180, −95.360479	BSLO 10-4	*Acinetobacter johnsonii strain 0183 311/359 (87%)*
12	Buffalo Bayou	29.765180, −95.360479	BSLO 20-1	*Pseudomonas putida KT2440 179/206 (87%)*
13	Buffalo Bayou	29.765180, −95.360479	BSLO 20-2	*Acinetobacter baumannii strain AZ-3443/515 (86%)*
14	Buffalo Bayou	29.765180, −95.360479	BSLO 20-3	*Acinetobacter junii strain Dg27 380/426 (89%)*
15	Carpenters Bayou	29.842077, −95.161694	CCLO 03	*Pseudomonas aeruginosa strain MDP-14 96/113 (85%)*
16	Carpenters Bayou	29.842077, −95.161694	CCLO 08	*Pseudomonas putida strain PM29 404/475 (85%)*
17	Carpenters Bayou	29.842077, −95.161694	CCLO 5-1	*Stenotrophomonas sp. Wr1C11 279/362 (77%)*
18	Carpenters Bayou	29.842077, −95.161694	CCLO 5-2	*[Pseudomonas] hibiscicola strain NAC65 288/377 (76%)*
19	Carpenters Bayou	29.842077, −95.161694	CCLO 5-3	*Brucella anthropi strain B8 16S 510/639 (80%)*
20	Carpenters Bayou	29.842077, −95.161694	CCLO 5-4	*Pseudomonas aeruginosa strain 0201761-1 281/303 (93%)*
21	Carpenters Bayou	29.842077, −95.161694	CCLO 5-5	*Pseudomonas chlororaphis subsp. piscium strain ChPhzS140 (77%)*
22	Carpenters Bayou	29.842077, −95.161694	CCLO 10-2	*Acinetobacter beijerinckii strain MI1 279/318 (88%)*
23	Carpenters Bayou	29.842077, −95.161694	CCLO 10-3	*Ruminococcus flavefaciens strain CCǪ-1 79/94 (84%)*
24	Carpenters Bayou	29.842077, −95.161694	CCLO 20-1	*Stenotrophomonas maltophilia strain SAM2 345/403 (86%)*
25	Carpenters Bayou	29.842077, −95.161694	CSLO 5-1	*Pseudomonas aeruginosa strain 0201761-1 288/337 (85%)*
26	Carpenters Bayou	29.842077, −95.161694	CSLO 10-1	*Uncultured Parvimonas sp. 123/154 (80%)*
27	Carpenters Bayou	29.842077, −95.161694	CSLO 10-2	*Brucella anthropi strain B8 540/699 (77%)*
28	Carpenters Bayou	29.842077, −95.161694	CSLO 20-3	*Pseudomonas mosselii strain 923 204/241 (85%)*

BCLO: Buffalo Bayou bacteria in clean lubricant oil, BSLO: Buffalo bacteria isolated in spent lubricant oil, CCLO: Carpenters Bayou bacteria isolated in clean lubricant oil, CSLO: Carpenters Bayou bacteria isolated in spent lubricant oil.

**Table 3:** *Kirby Bauer antibiotic susceptibility testing of various antimicrobial agents: S = susceptible, I = intermediate resistance, R = resistance. The zones of inhibition were mere measured by diameter (in mm) and interpreted based on the CLSI standards guidelines.* CLSI standards are provided for reference only and directly apply to *P. vulgaris*, an Enterobacteriaceae family member. In the case of the two *Pseduomonadaceae* family members, comparisons can be made between environmental isolates and their respective reference strains, while CLSI references presented in Table 3 do not directly apply to them.

Antibiotic	*Averg P. aeruginosa* Ref (Zone of Inhibition in mm)	*Averg P. aeruginosa* ENV (Zone of Inhibition in mm)	*Averg P. putida* Ref (Zone of Inhibition in mm)	*Averg P. putida* ENV *(Zone of Inhibition in mm)*	*Averg P. vulgaris* Ref *(Zone of Inhibition in mm)*	*Averg P. vulgaris* ENV (Zone of Inhibition in mm)	CLSI Standard		
							For Enteric Bacteria		
							(Zone Diameter mm)		
							S	I	R
Chloramphenicol 30 µg	10.0 ± 0.816	23.3 ± 0.943	10.7 ± 0.943	8.00 ± 0.816	11.3 ± 2.06	0.000	≥18	13–17	≤12
Tetracycline 30 µg	8.33 ± 1.25	0.000	14.0 ± 0.816	24.7 ± 1.25	15.7 ± 0.943	23.3 ± 1.25	≥19	15–18	≤14
Kanamycin 30 µg	0.067 ± 0.094	0.000	24.7 ± 0.943	0.000	24.0 ± 1.41	0.000	≥18	14–17	≤13
Penicillin 10 IU	0.133 ± 0.125	0.000	0.000	0.000	14.0 ± 1.63	0.000	≥22	12–21	≤11
Streptomycin 10 µg	0.033 ± 0.047	18.7 ± 0.943	18.3 ± 0.471	0.000	18.7 ± 0.943	0.000	≥15	12–14	≤11
Novobiocin 30 µg	0.033 ± 0.047	0.000	0.000	15.3 ± 0.471	9.33 ± 0.943	11.7 ± 0.943	≥22	18–21	≤17
Erythromycin 15 µg	0.067 ± 0.047	13.3 ± 0.471	0.000	23.7 ± 0.943	18.3 ± 2.36	12.7 ± 0.471	≥23	14–22	≤13
Neomycin 30 µg	0.033 ± 0.047	13.3 ± 1.25	20.7 ± 0.471	14.7 ± 0.471	20.7 ± 0.943	10.7 ± 0.943	≥17	13–16	≤12

## Data Availability

The original contributions presented in this study are included in the article. Further inquiries can be directed to the corresponding author.
